# The association of premorbid conditions with 6-month mortality in acutely admitted ICU patients over 80 years

**DOI:** 10.1186/s13613-024-01246-w

**Published:** 2024-03-30

**Authors:** Dylan W. de Lange, Ivo W. Soliman, Susannah Leaver, Ariane Boumendil, Lenneke E. M. Haas, Ximena Watson, Carol Boulanger, Wojciech Szczeklik, Antonio Artigas, Alessandro Morandi, Finn Andersen, Christian Jung, Rui Moreno, Sten Walther, Sandra Oeyen, Joerg C. Schefold, Maurizio Cecconi, Brian Marsh, Michael Joannidis, Yuriy Nalapko, Muhammed Elhadi, Jesper Fjølner, Bertrand Guidet, Hans Flaatten, the VIP2 study group, Philipp Eller, Raimund Helbok, René Schmutz, Joke Nollet, Nikolaas de Neve, Pieter De Buysscher, Walter Swinnen, Marijana Mikačić, Anders Bastiansen, Andreas Husted, Bård E S Dahle, Christine Cramer, Christoffer Sølling, Dorthe Ørsnes Christensen, Jakob Edelberg Thomsen, Jonas Juul Pedersen, Mathilde Hummelmose Enevoldsen, Thomas Elkmann, Agnieszka Kubisz-Pudelko, Alan Pope, Amy Collins, Ashok S Raj, Carole Boulanger, Christian Frey, Ciaran Hart, Clare Bolger, Dominic Spray, Georgina Randell, Helder Filipe, Ingeborg D Welters, Irina Grecu, Jane Evans, Jason Cupitt, Jenny Lord, Jeremy Henning, Joanne Jones, Jonathan Ball, Julie North, Kiran Salaunkey, Laura Ortiz-Ruiz De Gordoa, Louise Bell, Madhu Balasubramaniam, Marcela Vizcaychipi, Maria Faulkner, McDonald Mupudzi, Megan Lea-Hagerty, Michael Reay, Michael Spivey, Nicholas Love, Nick Spittle, Nick Spittle, Nigel White, Patricia Williams, Patrick Morgan, Phillipa Wakefield, Rachel Savine, Reni Jacob, Richard Innes, Ritoo Kapoor, Sally Humphreys, Steve Rose, Susan Dowling, Tarkeshwari Mane, Tom Lawton, Vongayi Ogbeide, Waqas Khaliq, Yolanda Baird, Antoine Romen, Arnaud Galbois, Christophe Vinsonneau, Cyril Charron, Didier Thevenin, Emmanuel Guerot, Guillaume Besch, Guillaume Savary, Hervé Mentec, Jean-Luc Chagnon, Jean-Philippe Rigaud, Jean-Pierre Quenot, Jeremy Castaneray, Jérémy Rosman, Julien Maizel, Kelly Tiercelet, Lucie Vettoretti, Maud Mousset Hovaere, Messika Messika, Michel Djibré, Nathalie Rolin, Philippe Burtin, Pierre Garcon, Saad Nseir, Xavier Valette, Christian Rabe, Eberhard Barth, Henning Ebelt, Kristina Fuest, Marcus Franz, Michael Horacek, Michael Schuster, Patrick Meybohm, Raphael Romano Bruno, Sebastian Allgäuer, Simon Dubler, Stefan J Schaller, Stefan Schering, Stephan Steiner, Thorben Dieck, Tim Rahmel, Tobias Graf, Anastasia Koutsikou, Aristeidis Vakalos, Bogdan Raitsiou, Elli Niki Flioni, Evangelia Neou, Fotios Tsimpoukas, Georgios Papathanakos, Giorgos Marinakis, Ioannis Koutsodimitropoulos, Kounougeri Aikaterini, Nikoletta Rovina, Stylliani Kourelea, Tasioudis Polychronis, Vasiiios Zidianakis, Vryza Konstantinia, Zoi Aidoni, Catherine Motherway, Chris Read, Ignacio Martin-Loeches, Andrea Neville Cracchiolo, Aristide Morigi, Italo Calamai, Stefania Brusa, Ahmed Elhadi, Ahmed Tarek, Ala Khaled, Hazem Ahmed, Wesal Ali Belkhair, Alexander D Cornet, Diederik Gommers, Dylan de Lange, Eva van Boven, Jasper Haringman, Lenneke Haas, Lettie van den Berg, Oscar Hoiting, Peter Jager, Rik T Gerritsen, Tom Dormans, Willem Dieperink, Alena Breidablik, Alena Breidablik, Anita Slapgard, Anne-Karin Rime, Bente Jannestad, Britt Sjøbøe, Eva Rice, Finn H Andersen, Hans Frank Strietzel, Jan Peter Jensen, Jørund Langørgen, Kirsti Tøien, Kristian Strand, Michael Hahn, Pål Klepstad, Aleksandra Biernacka, Anna Kluzik, Bartosz Kudlinski, Dariusz Maciejewski, Dorota Studzińska, Hubert Hymczak, Jan Stefaniak, Joanna Solek-Pastuszka, Joanna Zorska, Katarzyna Cwyl, Lukasz J Krzych, Maciej Zukowski, Małgorzata Lipińska-Gediga, Marek Pietruszko, Mariusz Piechota, Marta Serwa, Miroslaw Czuczwar, Mirosław Ziętkiewicz, Natalia Kozera, Paweł Nasiłowski, Paweł Sendur, Paweł Zatorski, Piotr Galkin, Ryszard Gawda, Urszula Kościuczuk, Waldemar Cyrankiewicz, Wojciech Gola, Alexandre Fernandes Pinto, Ana Margarida Fernandes, Ana Rita Santos, Cristina Sousa, Inês Barros, Isabel Amorim Ferreira, Jacobo Bacariza Blanco, João Teles Carvalho, Jose Maia, Nuno Candeias, Nuno Catorze, Vladislav Belskiy, Africa Lores, Angela Prado Mira, Catia Cilloniz, David Perez-Torres, Emilio Maseda, Enver Rodriguez, Estefania Prol-Silva, Gaspar Eixarch, Gemma Gomà, Gerardo Aguilar, Gonzalo Navarro Velasco, Marián Irazábal Jaimes, Mercedes Ibarz Villamayor, Noemí Llamas Fernández, Patricia Jimeno Cubero, Sonia López-Cuenca, Teresa Tomasa, Anders Sjöqvist, Camilla Brorsson, Fredrik Schiöler, Henrik Westberg, Jessica Nauska, Joakim Sivik, Johan Berkius, Karin Kleiven Thiringer, Lina De Geer, Filippo Boroli, Joerg C Schefold, Leila Hergafi, Philippe Eckert, Ismail Yıldız, Ihor Yovenko, Richard Pugh

**Affiliations:** 1 Department of Intensive Care Medicine, University Medical Center, University Utrecht, Heidelberglaan 100, 3584 CX Utrecht, The Netherlands; 2 Department of Intensive Care Medicine, University Medical Center, University Utrecht, Utrecht, The Netherlands; 3 Department of critical care, St George’s Hospital London, London, UK; 4 AP-HP, Hôpital Saint-Antoine, service de reanimation, F75012 Paris, France; 5 Department of Intensive Care, Diakonessen Hospital, Utrecht, The Netherlands; 6 Intensive Care Unit, Royal Devon & Exeter NHS Foundation Trust, Exeter, UK; 7 Center for Intensive Care and Perioperative Medicine, Jagiellonian University Medical College, Kraków, Poland; 8 Department of Intensive Care Medecine, CIBER Enfermedades Respiratorias, Corporacion Sanitaria Universitaria Parc Tauli, Autonomous University of Barcelona, Sabadell, Spain; 9 Critical Care Department, Sagrado Corazon-General de Cataluña University Hospitals, Quiron Salud, Barcelona, Spain; 10 Department of Rehabilitation Hospital Ancelle di Cremona, Cremona, Italy; 11 Geriatric Research Group, Brescia, Italy; 12 Department of Anaesthesia and Intensive Care, Ålesund Hospital, Ålesund, Norway; 13 NTNU, Department of Circulation and Medical Imaging, Trondheim, Norway; 14 Division of Cardiology, Pulmonology and Vascular Medicine, University Hospital Düsseldorf, Heinrich-Heine-University, Düsseldorf, Germany; 15 Faculdade de Ciências Médicas de Lisboa (Nova Médical School), Hospital de São José, Centro Hospitalar Universitário de Lisboa Central, Lisbon, Portugal; 16 Faculdada de Ciências de Saúde, Universidade da Beira Interior, Covilhã, Portugal; 17 Linkoping University Hospital, Linkoping, Sweden; 18 Department of Intensive Care 1K12IC, Ghent University Hospital, Ghent, Belgium; 19 Department of Intensive Care Medicine, Inselspital, Universitätsspital, University of Bern, Bern, Switzerland; 20 Department of Anesthesia and Intensive Care Medicine, Humanitas Clinical and Research Center - IRCCS, Via Alessandro Manzoni, 56, 20089 Rozzano, MI Italy; 21 Department of Biomedical Sciences, Humanitas University, Pieve Emanuele, MI Italy; 22 Mater Misericordiae University Hospital, Dublin, Ireland; 23 Division of Intensive Care and Emergency Medicine, Department of Internal Medicine, Medical University Innsbruck, Innsbruck, Austria; 24 European Wellness International, ICU, Luhansk, Ukraine; 25 Alkhums Hospital, ICU, Tripoli, Libya; 26 Department of Anaesthesia and Intensive Care, Viborg Regional Hospital, Viborg, Denmark; 27 Sorbonne Université, INSERM, Institut Pierre Louis d’Epidémiologie et de Santé Publique, AP-HP, Hôpital Saint-Antoine, service de reanimation, 75012 Paris, France; 28 Department of Clinical Medicine, Department of Anaesthesia and Intensive Care, University of Bergen, Haukeland University Hospital, Bergen, Norway

**Keywords:** Critical care, Outcome, Frailty, Cognitive functioning, Activities of daily living, Comorbidity

## Abstract

**Background:**

Premorbid conditions influence the outcome of acutely ill adult patients aged 80 years and over who are admitted to the ICU. The aim of this study was to determine the influence of such premorbid conditions on 6 month survival.

**Methods:**

Prospective cohort study in 242 ICUs from 22 countries including patients 80 years or above, admitted over a 6 months period to an ICU between May 2018 and May 2019. Only emergency (acute) ICU admissions in adult patients ≥ 80 years of age were eligible. Patients who were admitted after planned/elective surgery were excluded. We measured the Clinical Frailty Scale (CFS), the Informant Questionnaire on Cognitive Decline in the Elderly (IQCODE), disability with the Katz activities of daily living (ADL) score, comorbidities and a Polypharmacy Score (CPS).

**Results:**

Overall, the VIP2 study included 3920 patients. During ICU stay 1191 patients died (30.9%), and another 436 patients (11.1%) died after ICU discharge but within the first 30 days of admission, and an additional 895 patients died hereafter but within the first 6 months after admission (22.8%). The 6 months mortality was 64%. The median CFS was 4 (IQR 3–6). Frailty (CFS ≥ 5) was present in 26.6%. Cognitive decline (IQCODE above 3.5) was found in 30.2%. The median IQCODE was 3.19. A Katz ADL of 4 or less was present in 27.7%. Patients who surviving > 6 months were slightly younger (median age survivors 84 with IQR 81–86) than patients dying within the first 6 months (median age 84, IQR 82–87, *p* = 0.013), were less frequently frail (CFS > 5 in 19% versus 34%, *p* < 0.01) and were less dependent based on their Katz activities of daily living measurement (median Katz score 6, IQR 5–6 versus 6 points, IQR 3–6, *p* < 0.01).

**Conclusions:**

We found that Clinical Frailty Scale, age, and SOFA at admission were independent prognostic factors for 6 month mortality after ICU admission in patients age 80 and above. Adding other geriatric syndromes and scores did not improve the model. This information can be used in shared-decision making.

*ClinicalTrials.gov*: NCT03370692.

**Supplementary Information:**

The online version contains supplementary material available at 10.1186/s13613-024-01246-w.

## Background

Currently, more than 15% of patients admitted to intensive care units (ICUs) are 80 years of age or older. This proportion of “very old intensive care patients” (VIPs) is estimated to increase to 36% by 2025 [1–3]. This has been identified as a public health challenge, because patients aged 80 years and older consume a large proportion of healthcare resources and budgets, while mortality rates are consistently reported to be higher than in younger patients. This is particularly true for older adult patients aged 80 years and over who are admitted acutely [4]. For unplanned admissions, the overall 30-day survival rate for ICU patients is approximately 60% [5, 6].

However, looking at short-term prognosis may be of limited value, as patients usually aim for long-term survival with a good quality of life (QoL) [7]. Intensive care may even be perceived as disproportionate if it only causes suffering and anxiety without achieving the goal of long-term survival. It is, therefore, of paramount importance to better identify elderly patients who are likely to have a good long-term outcome. Previous reports have found an association between pre-morbid conditions and 30 day survival [6]. In very old adult patients, the ability to cope with severe stressors such as critical illness appears to be related to geriatric syndromes such as frailty [5, 6], cognitive decline [8, 9] and reduced performance on the Activities of Daily Living (ADL) scale, in addition to comorbidity. However, the impact of these premorbid conditions on the long-term outcome of acutely admitted elderly ICU patients has not been established. Does the severity of illness on admission to the ICU or premorbid conditions predict a patient's chance of long-term survival?

In this European-based study, we aimed to examine the influence of three common geriatric syndromes: frailty, cognitive impairment and disability, and the presence of comorbidity and polypharmacy, and to assess their influence on 6 month survival.

## Methods

### Design and setting

This was a prospective observational study in 242 ICUs from 22 European countries, including Turkey. The inclusion period was from May 2018 to May 2019. Most patients were included in the winter of 2018–2019. The 6 month follow-up period ended on 1 December 2019.

The study was coordinated by the Health Services Resource and Outcome (HSRO) and Nurses and Allied Health care Professionals (NAHP) sections of the European Society of Intensive Care Medicine (ESICM). Each country had a national coordinator responsible for ICU recruitment and application for national or regional ethical and regulatory approval of the study. Institutional research ethics committee approval was obtained at each study site. Individual ICUs were asked to enrol consecutive patients for 6 months during the 1 year study period and were allowed to stop when ≥ 20 patients had been enrolled. Participation in the 6 month follow-up was optional for the ICUs. A dedicated website was set up to facilitate information about the study and its progress, and to allow data entry using an electronic case record form (eCRF). The trial is registered at www.ClinicalTrials.gov (ID: NCT03370692). There was no specific funding for this study or for the participating ICUs (Additional file 1).

### Participants

Only emergency (acute) ICU admissions of adult patients aged ≥ 80 years were eligible. Patients admitted after planned/elective surgery were excluded, because their mortality rates are completely different (lower) than those of acutely admitted very old critically ill patients [4].

### Data collection

#### Data collection at admission

For each eligible patient, demographic data were collected: age, sex, place of residence prior to hospital admission, and reason for admission according to a predefined list (see Additional file 3). Second, the study collected mandatory data on the patient’s geriatric conditions prior to this hospital admission, including the Clinical Frailty Scale (CFS) [10]. For the assessment of frailty, we defined the level of frailty present prior to hospital admission and unaffected by the current acute illness. The information needed to make this assessment was provided by the patient or proxy, or obtained from the patient’s medical record. The profession of the assessor was documented. The simple description of CFS was used with permission [10]. Frail patients were defined as having a CFS of ≥ 5. We also recorded Katz activities of daily living (Katz ADL) [11], with an ADL score ≤ 4 defining disability. Pre-admission cognitive function was assessed using the Short form of Informant Questionnaire on Cognitive Decline in the Elderly (IQCODE) [12]. The information used to calculate the IQCODE was provided by caregivers who had known the patient well for the previous 10 years. We defined cognitive decline as an IQCODE > 3.5 [12]. A comorbidity and polypharmacy score (CPS) was calculated [13]. The CPS was defined as the simple sum of the number of known comorbidities and the number of different medications taken daily before admission (1 point for each chronic comorbidity and 1 point for each medication). Cardiovascular dysfunction was counted per morbidity (e.g., a patient with hypertension, atrial fibrillation and congestive heart failure would receive 3 points, even if they were all cardiovascular comorbidities). The number can vary from 0 (no comorbidity, no medication) to infinity, although in most patients the number was < 20. The severity of CPS has traditionally been stratified as minor/mild (CPS 0–7), moderate (8–14) and severe (≥ 15). In the analyses, the total CPS score was used for analyses (Additional files 4, 5, 6).

#### Data collection of variables during ICU admission

The Sequential Organ Failure Assessment (SOFA) score was calculated within the first 24 h of ICU admission.14 The total SOFA score on admission was calculated using an online calculator in the eCRF. Length of stay (LOS) in the ICU was recorded as the number of hours between admission and discharge and later converted to days (consecutive 24 h periods rather than calendar days) for analysis. Any period of non-invasive or invasive (with endotracheal intubation or tracheostomy) mechanical ventilation, use of vasoactive drugs and renal replacement therapy (RRT) was recorded with the start (which ICU treatment day) and duration of the procedure (in hours).

Limitation of life-sustaining therapies (LST), such as withholding or withdrawing life-sustaining treatments, was documented when applied and the timing of LST limitation was recorded (in days since ICU admission) [15].

Outcome was assessed as survival at ICU discharge, 30 days and 6 months (180 days) after ICU admission. The source of information for vital status at 6 months was documented.

The CRF and database were hosted on a secure server on the campus of Aarhus University, Denmark.

#### Bias

The ICUs were asked to include all consecutive patients ≥ 80 years that were acutely admitted to the ICU, irrespective of the anticipated duration of ICU stay.

#### Study size

We had no formal calculation in this purely observational study. We estimated the 6 month mortality to be approximately 50%, as seen in similar study populations [5, 6].

#### Data imputation

We used multiple imputation of data to compensate for missing values in both predictors and outcomes. In short, missing data were imputed to create 100 different data sets, in which we used 50 iterations to achieve value stability. Non-linear models were used and variable order and predictors were set to avoid feedback loops. Post-processing was set to limit extreme values in continuous variables. Rubin’s rules were used to pool the results of the final analysis. Further details of the methods have been described in previously published papers [5, 16].

#### Statistical analysis

Baseline characteristics of patients were analyzed as frequencies and percentages for categorical variables and as medians and interquartile ranges (IQRs) for continuous variables.

After multiple imputations we used these datasets to calculate the area-under-the-curve (AUC) for various geriatric variables that were available premorbid to predict 6 month outcome:

- age of the patient at admission to the ICU
- the CFS as determined prior to this disease episode.
- the cumulative CPS score as determined at admission to the ICU
- the Katz ADL
- the SOFA-score at admission to the ICU

All analyses were performed with R software, version 3.2.2 (R Foundation for Statistical Computing).

## Results

### Participants

There were 242 participating ICUs in the 22 European countries including Turkey. The characteristics of these ICUs are listed in the electronic Additional file and recruitment per country is presented in Additional file 1.

A total of 3920 patients were enrolled in the VIP2 study. The demographics of these patients are shown in Tables 1 and 2. The median age of participants was 84 years (IQR 81–86), 53% were male, the median number of chronic comorbidities was 4 (IQR 3–4) and the median number of different medications taken daily was 6 (IQR 4–9). This resulted in a median CPS of 10 (IQR 7–14). 61% of patients had a CFS > 3, meaning that they were either pre-frail or frail at the time of ICU admission. The median SOFA score was 6 (IQR 6–9).

**Table 1:** Patient characteristics of the total study population, the patients of whom all information was available in the database (complete cases), the patients with missing data (incomplete cases) and the differences between these two groups

Patient characteristic [*n* missing]	Total population	Complete cases	Incomplete cases	*p* value for difference between complete and incomplete cases
N	3920	2855	1065	
Age [0]	84 [81–87]	84 [81–87]	84 [81–86]	0.738
Sex [0]	2051 (53%)	1532 (54%)	519 (51%)	0.072
IQCODE [922]	3 [3, 4]	3 [3, 4]	3 [3, 4]	0.681
Chronic comorbidities [6]	4 [3–6]	4 [3–6]	4 [3–6]	0.703
Drugs daily [7]	6 [4–9]	6 [4–9]	6 [4–9]	0.73*2*
CPS [7]	10 [7–14]	10 [7–14]	11 [7–14]	0.633
CFS 1–3 [17]	1544 (39%)	1170 (41%)	374 (35%)	0.011
CFS 4–5	1314 (34%)	936 (33%)	378 (35%)	
CFS 6–9	1045 (27%)	749 (26%)	296 (28%)	
Katz [433]	6 [4–6]	6 [4–6]	6 [4–6]	0.952
SOFA [11]	6 [4–9]	6 [4–9]	6 [3–9]	0.94
intubated [0]	1943 (50%)	1429 (50%)	514 (50%)	0.941
tracheostomy [10]	264 (7%)	201 (7%)	63 (6%)	0.37
vasoactive medication [0]	2326 (59%)	1741 (61%)	585 (57%)	0.019
RRT [0]	424 (11%)	338 (12%)	86 (8%)	0.002
NIV [0]	881 (23%)	669 (24%)	212 (21%)	0.065
withhold [0]	1140 (29%)	893 (31%)	246 (23%)	< 0.001
withdraw [0]	545 (14%)	404 (14%)	141 (14%)	0.746
survived to icu discharge [14]	2729 (72%)	2033 (72%)	759 (74%)	0.06
survived up to 30 days [74]	2293 (58%)	1648 (58%)	545 (64%)	< 0.001
survived up to 6 months [644]	1398 (36%)	1345 (47%)	53 (6%)	< 0.001

IQCODE means the Short form of Informant Questionnaire on Cognitive Decline in the Elderly, chronic comorbidities means the number of chronic comorbidities at admission to the ICU, drugs daily means the amount of drugs taken daily, CPS means the comorbidities and poly pharmacy score (which is the combination of drugs daily and chronic comorbidities), CFS means the clinical frailty scale, Katz means the Katz activities of daily living score, SOFA means the sequential organ failure assessment score, RRT means renal replacement therapy during ICU stay, NIV means non-invasive ventilation during ICU stay

**Table 2:** Differences in patients surviving up to 30 days versus 6 months (complete case data)

	All patients (*n* = 3920)	Survivors at 30 days (*n* = 2293)	Survivors at 6 months (*n* = 1398)	Deceased at 6 months (*n* = 1825)	*p* value
N [N missing]	3867	2293	1398	1825	
Age [0]	84 [81–87]	84 [81–87]	84 [81–86]	84 [82–87]	0.013
Gender [0]	2051 (53%)	1185 (52%)	731 (52%)	1007 (55%)	0.111
IQCODE [922]	3 [3, 4]	3 [3, 4]	3 [3]	3 [3, 4]	0
Chronic comorbidities [6]	4 [3–6]	4 [3–5]	4 [3–5]	4 [3–6]	0.002
Drugs daily [7]	6 [4–9]	6 [4–9]	6 [4–8]	6 [4–9]	0.04
CPS [7]	10 [7–14]	10 [7–14]	10 [7–14]	11 [7–14]	0.006
CFS 1–3 [17]	1532 (40%)	999 (44%)	658 (47%)	620 (34%)	< 0.01
CFS 4–5 [17]	1284 (33%)	795 (35%)	476 (34%)	574 (31%)	
CFS 6–9 [17]	1034 (27%)	496 (22%)	263 (19%)	616 (34%)	
Katz [433]	6 [4–6]	6 [5, 6]	6 [5, 6]	6 [3–6]	< 0.01
SOFA [11]	6 [4–9]	5 [3–8]	5 [3–7]	8 [5–10]	< 0.01
Intubated [0]	1943 (50%)	932 (41%)	512 (37%)	1153 (63%)	< 0.01
Tracheostomy [10]	264 (7%)	168 (7%)	65 (5%)	157 (9%)	< 0.01
vasoactive medication [0]	2326 (60%)	1169 (51%)	699 (50%)	1324 (73%)	< 0.01
RRT [0]	424 (11%)	163 (7%)	92 (7%)	295 (16%)	< 0.01
NIV [0]	881 (23%)	538 (23%)	330 (24%)	400 (22%)	0.275
withhold [0]	1139 (29%)	389 (17%)	222 (16%)	830 (45%)	< 0.01
withdraw [0]	545 (14%)	27 (1%)	13 (1%)	526 (29%)	< 0.01

IQCODE means the Short form of Informant Questionnaire on Cognitive Decline in the Elderly, chronic comorbidities means the number of chronic comorbidities at admission to the ICU, drugs daily means the amount of drugs taken daily, CPS means the comorbidities and poly pharmacy score (which is the combination of drugs daily and chronic comorbidities), CFS means the clinical frailty scale, Katz means the Katz activities of daily living score, SOFA means the sequential organ failure assessment score, RRT means renal replacement therapy during ICU stay, NIV means non-invasive ventilation during ICU stay

During their stay in the ICU, 1191 patients (30.9%) died, a further 436 patients (11.1%) died after discharge from the ICU but within the first 30 days of admission, and a further 895 patients died afterwards but within the first 6 months of admission (22.8%). After 6 months, 64% of patients had died. The characteristics of the surviving patients are shown in Table 2. These figures are derived from the complete data set.

Patients who survived up to 6 months were slightly younger (median age of survivors 84, IQR 81–86) than those who died within the first 6 months (median age 84, IQR 82–87, *p* = 0.013), less likely to be frail (CFS > 5 in 19% versus 34%, *p* < 0.01) and less dependent on their Katz ADL (median Katz score 6, IQR 5–6 versus 6 points, IQR 3–6, *p* < 0.01).

### Outcome data

**Fig. 1:**
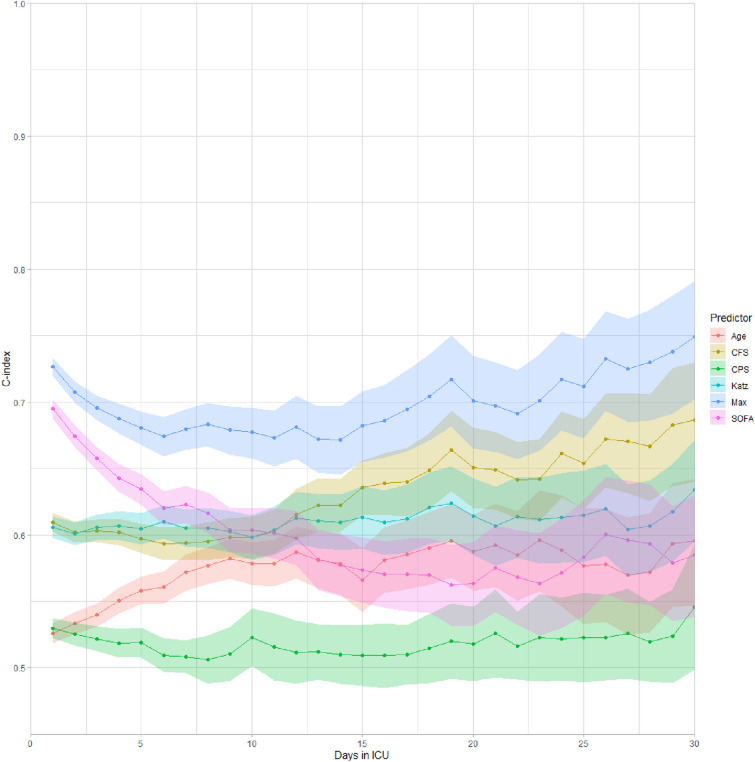
Predictive value of various patients characteristics on 6 month mortality expressed by the area-under-the-curve over time. The SOFA score has the highest association with mortality in the first 6 days after admission to the ICU for an acute reason (pink line and confidence interval). However, after approximately 12 days the AUC of the CFS (brown line) was higher than the AUC of the SOFA-score, which means that CFS, from that point onwards, is more associated with 6 month outcome. The blue line with the highest AUC is all variables combined (age, CFS, CPS, Katz and SOFA)

The relationship between the geriatric variables available before the current period of illness (premorbid) and the scores for the predictability of death in the first 6 months after ICU admission is shown in Fig. 1. The C-statistic (area under the curve of the sensitivity and specificity calculations) varies over time. The full model consisting of all variables (age, SOFA score, frailty, CPS and Katz) had the highest AUC for the outcome of death at 6 months. Note that the AUC for frailty associated with death at 6 months had an upward slope. After 12 days in the ICU, CFS on admission has a higher C-statistic for 6 month mortality than SOFA score on admission.

## Discussion

We have shown that the 6 month survival rate for patients aged 80 years and older after acute admission to the ICU is 36%. Survival up to 6 months depends on several factors, which can be divided into factors associated with severity of illness (SOFA score) or factors that are patient-dependent (such as age, frailty, cognitive function and independence in activities of daily living). Severity can be modified by treatments administered in the ICU, but premorbid patient characteristics are fixed and cannot be modified by interventions in such acute settings.

In this study, we showed that when patients aged ≥ 80 years who were acutely admitted to the ICU were alive for more than 12 days, the Clinical Frailty Scale (CFS) model outperformed the SOFA score model and the age-based model in predicting 6 month mortality. Therefore, for patients who have survived their acute critical illness (the severity of illness associated with the reason for admission, e.g. trauma or infection), long-term survival is now more dependent on their ability to fully recover. Obviously, this is inversely related to the level of comorbidity and frailty of the patient.

The impact of frailty on 6 month mortality increases over time (represented by the increasing C-statistic during the 30 days in the ICU). Frailty, defined as CFS 6–9, was associated with 6 month survival of 31.9%, compared with 53.9% for fit patients (CFS 1–3) and 47.4% for frail patients (CFS 4–5) (*p* < 0.01). Frailty is associated with an inability to cope with the physical stress associated with acute critical illness. Once the acute effects of the illness have subsided, the patient is left with further organ damage that they are unable to cope with. This results in very high mortality rates during and after ICU treatment.

In shared decision making with patients or their families, one of the key questions is: "What are the long-term chances of being alive? This question is particularly important for adult patients aged 80 and over. Their chances of long-term survival are already limited because of their advanced age. In addition, their chances of survival decrease significantly if they require acute hospitalisation for a serious illness [4]. Here we have shown an overall survival of 36% after 6 months. This low survival in the first 6 months after ICU admission is in line with previous publications [17–21] and remains higher than in an age- and sex-matched population in the first year after ICU discharge [22].

We have also shown that long-term survival is inversely associated with pre-morbid conditions (age and frailty) and the severity of illness at admission. Unfortunately, many of these variables are not amenable to intervention. As frustrating as this may be, it also shows that we can inform our patients (or, more likely, surrogate decision makers) about possible outcomes early in the course of the disease. While it is impossible to predict complete futility of treatment, we can explain to surrogate decision makers what a patient's chances are based on their premorbid conditions [23]. Such information is of paramount importance in shared decision making. Interestingly, cognitive impairment and age alone were only weakly associated with 6 month survival.

### Limitation of this study

Older patients discharged from the ICU often have lower functional capacity and independence than before admission to the ICU. In a cohort of 610 Canadian patients aged ≥ 80 years admitted to an ICU, only 26% were alive at 12 months and had recovered their baseline physical function [24]. This is particularly important as many older patients prioritise quality of life over longevity [7]. Indeed, the majority of patients (74%) reported that they would not choose treatment if the burden of that treatment was high and the expected outcome was survival with severe functional impairment [25, 26]. When community-dwelling older people (average age 85 years) were well informed about potential ICU treatments by watching videos of what this treatment would actually look like, many chose not to undergo such treatments [26]. This clearly shows that survival alone is not enough to fully inform patients or surrogate decision makers. Estimated quality of life, functional independence and autonomy may be more important in this age group. These outcomes are missing from our study and are a major limitation.

Another limitation could be ‘admission bias’; we did not record the reasons or outcomes of patients who were refused ICU admission. Therefore, we may be looking at a selected population that may have a better outcome than all patients aged 80 and over.

Another limitation of our study is that “advanced directives” and/or “restrictions on life-sustaining treatment” can have a huge impact on outcome. Here we see a self-fulfilling prophecy. We see reduced survival in patients for whom physicians expect limited survival and have discussed limitations on life-sustaining treatments. We have previously reported that frail patients have more restrictions on life-sustaining treatments [15].

Another limitation is that 6 month survival data were not available for all patients. We cannot exclude that patients with missing 6 month survival data were different from those included in the study.

Finally, we did not collect information on other explanatory variables, such as individual socio-economic status, education, nutritional status, lack of delirium assessment on admission and severity of illness later during the ICU stay. These variables are important for future research.

### Strong feature of this study

A strong feature of this study is that we prospectively examined outcomes in a well-defined group of consecutive, acutely admitted, elderly patients over 80 years of age in 20 European countries. The results are, therefore, valid for a large proportion of these older patients (external validity).

## Conclusions

We found that geriatric conditions that were present before a patient's acute illness (so-called premorbid conditions) were associated with the likelihood of survival. Apart from the SOFA score, which reflects acute illness, the Clinical Frailty Scale and age were independent prognostic factors for 6 month mortality after ICU admission in patients aged 80 years and older. The addition of other geriatric syndromes and scores did not improve this association with mortality. Knowledge of these premorbid conditions is important information that can be used to guide decisions about both the benefits of ICU admission and the benefits of continued ICU treatment as part of the shared decision-making process with the patient, family, or surrogate decision-makers.

### Supplementary Information

**Additional file 1.** Participating ICUs and countries.

**Additional file 2.** Inclusion period: number of patients included per week.

**Additional file 3.** Recorded study variables.

**Additional file 4.** Clinical frailty scale (CFS).

**Additional file 5.** Katz activity of daily living (ADL).

**Additional file 6.** Cognitive decline questionnaire (IQCODE).

## Data Availability

The data sets used and/or analysed during the current study are available from the corresponding author on reasonable request.

## Abbreviations

CFS: Clinical frailty scale
CPS: Comorbidity and polypharmacy score
ICU: Intensive care unit
IQCODE: Informant questionnaire on cognitive decline in the elderly
SOFA: Sequential organ failure assessment
