# Supplementary material for: The association of premorbid conditions with 6-month mortality in acutely admitted ICU patients over 80 years
Source: Ann Intensive Care. 2024 Mar 30;14:46. doi: 10.1186/s13613-024-01246-w (PMC10981642; doi:10.1186/s13613-024-01246-w)
Supplement: Supplementary file 1 — Additional file 1. Participating ICUs and countries. [file 13613_2024_1246_MOESM1_ESM.docx]

**ESM1: Participating ICUs and countries**

The numbers represent: Numbers of ICUs/Sum of recruited patients in each country

**
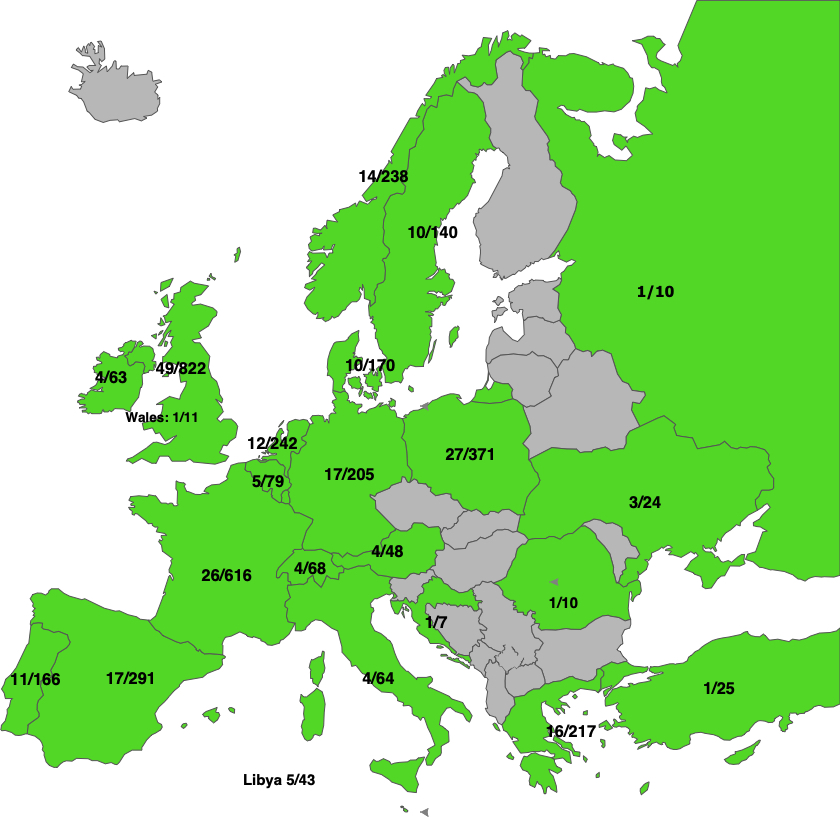
**
