# Supplementary material for: The association of premorbid conditions with 6-month mortality in acutely admitted ICU patients over 80 years
Source: Ann Intensive Care. 2024 Mar 30;14:46. doi: 10.1186/s13613-024-01246-w (PMC10981642; doi:10.1186/s13613-024-01246-w)
Supplement: Supplementary file 2 — Additional file 2. Inclusion period: number of patients included per week. [file 13613_2024_1246_MOESM2_ESM.docx]

**ESM2:** Inclusion period: number of patients included per week.

The x axis means the number of the week starting week 18 of year 2018 and ending week 22 of year 2019.

**
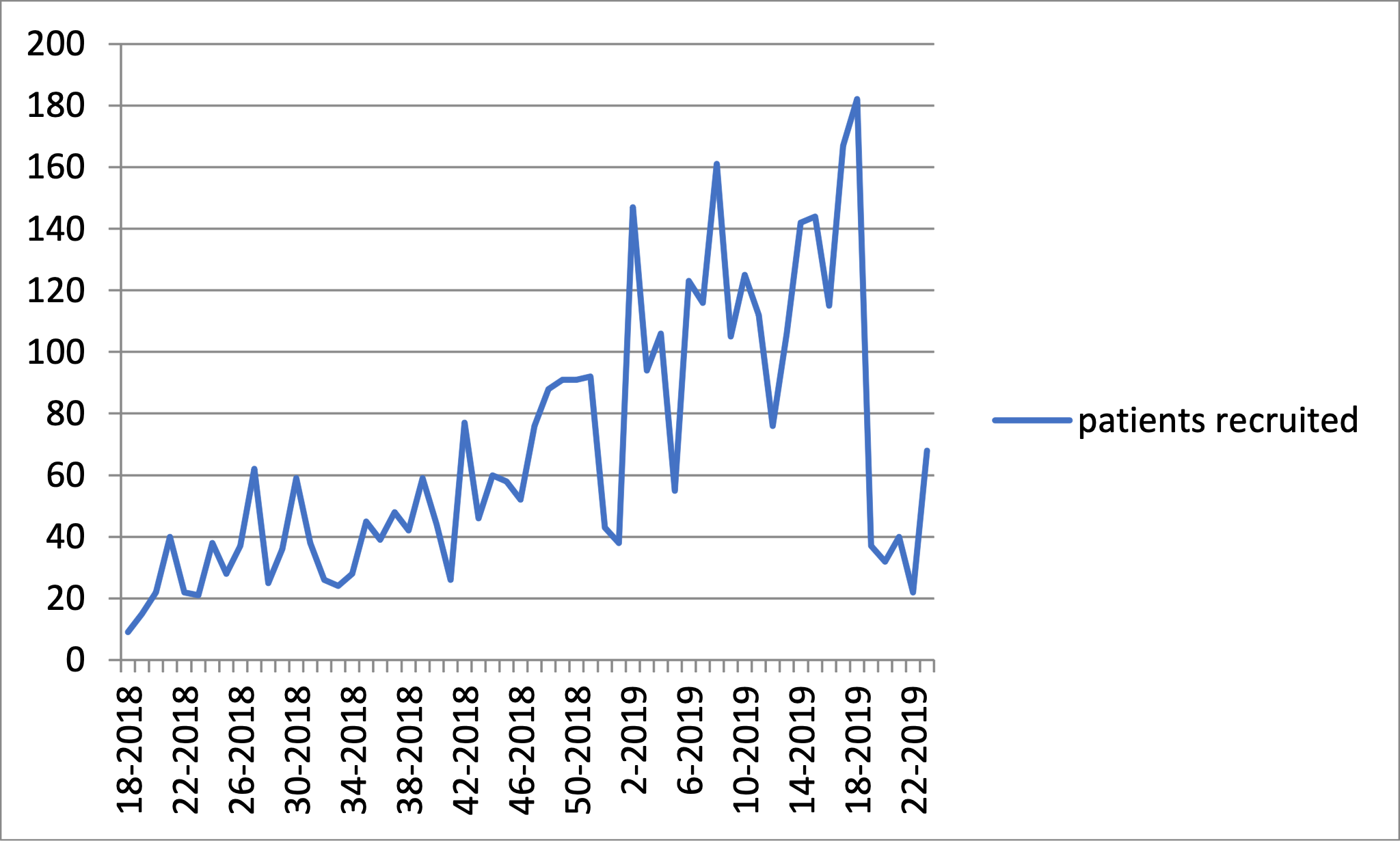
**
