# Supplementary material for: The association of premorbid conditions with 6-month mortality in acutely admitted ICU patients over 80 years
Source: Ann Intensive Care. 2024 Mar 30;14:46. doi: 10.1186/s13613-024-01246-w (PMC10981642; doi:10.1186/s13613-024-01246-w)
Supplement: Supplementary file 3 — Additional file 3. Recorded study variables. [file 13613_2024_1246_MOESM3_ESM.docx]

**ESM3 :** Recorded study variables

| **Variable** | **Definition** | **Comments** |
| --- | --- | --- |
| Age | Years | Whole number ≥80 |
| Gender | Male/Female |  |
| Place of living before ICU admission | Own home (including if with spouse)  Other home with family or caregivers  Nursing home  Hospital ward  Other |  |
| Reason for ICU admission | Respiratory failure Circulatory failure  Combined respiratory /circulatory failure  Severe sepsis (according to Sepsis 3)  Multitrauma without head injury  Multitrauma with head injury Isolated head injury Intoxication  Non-traumatic cerebral pathology  Emergency surgery  Other causes | Only one to be selected (best suited) |
| Sequential organ failure assessment (SOFA) score | Value 0 to 4 in six vital organ systems | Range 0-24 |
| Clinical Frailty Scale (CFS) | Pictogram: Values from 1 to 9 | If participating in the interrater variability the data was entered twice |
| Assessors profession | ICU Nurse  ICU physician  Dedicated research staff  Other |  |
| Information obtained from | Patient  Family/Care-givers  Hospital records  Other |  |
| Informant-questionnaire-on-cognitive-decline-in-the-elderly (IQCODE) | Was performed  Was not possible  Was possible but not performed | 16 questions about cognition compared to 10 years ago |
| IQCODE value | Average of 16 items | 1 to 5 |
| KATZ index of independence in activities of daily living (ADL) | Was performed  Was not possible  Was possible but not performed | 6 assessments from 0 to 1 |
| KATZ ADL value | Sum of assessments | 0-6 points |
| Co-morbidity and polypharmacy score (CPS) | Sum of comorbidities and number of daily drugs | 0 To infinitely |
| **ICU interventions** |  |  |
| Intubation | Yes/No | Start (ICU day) and duration (h) |
| Vasoactive drugs | Yes/No | Start (ICU day) and duration (h) |
| Noninvasive ventilation | Yes/No | Start (ICU day) and duration (h) |
| Renal Replacement therapy (RRT) | Yes/No | Start (ICU day) and duration (h) |
| Tracheostomy performed | Yes/no |  |
| Treatment withheld | Yes/No | ICU day |
| Treatment withdrawn | Yes/No | ICU day |
| ICU LOS | hours |  |
| Survived to ICU discharge | Yes/no |  |
| Survived 30 days | Yes/No | If no Day of death after |
| How was survival assessed: | Hospital files or patient record,  Direct contact (phone, mail) to patient/relatives,  Direct contact (phone, mail) to general practitioner,  National statistics registry, Municipal personal records Other/unknown |  |
