# Supplementary material for: The association of premorbid conditions with 6-month mortality in acutely admitted ICU patients over 80 years
Source: Ann Intensive Care. 2024 Mar 30;14:46. doi: 10.1186/s13613-024-01246-w (PMC10981642; doi:10.1186/s13613-024-01246-w)
Supplement: Supplementary file 4 — Additional file 4. Clinical frailty scale (CFS). [file 13613_2024_1246_MOESM4_ESM.docx]

**ESM4: Clinical frailty scale (CFS)**


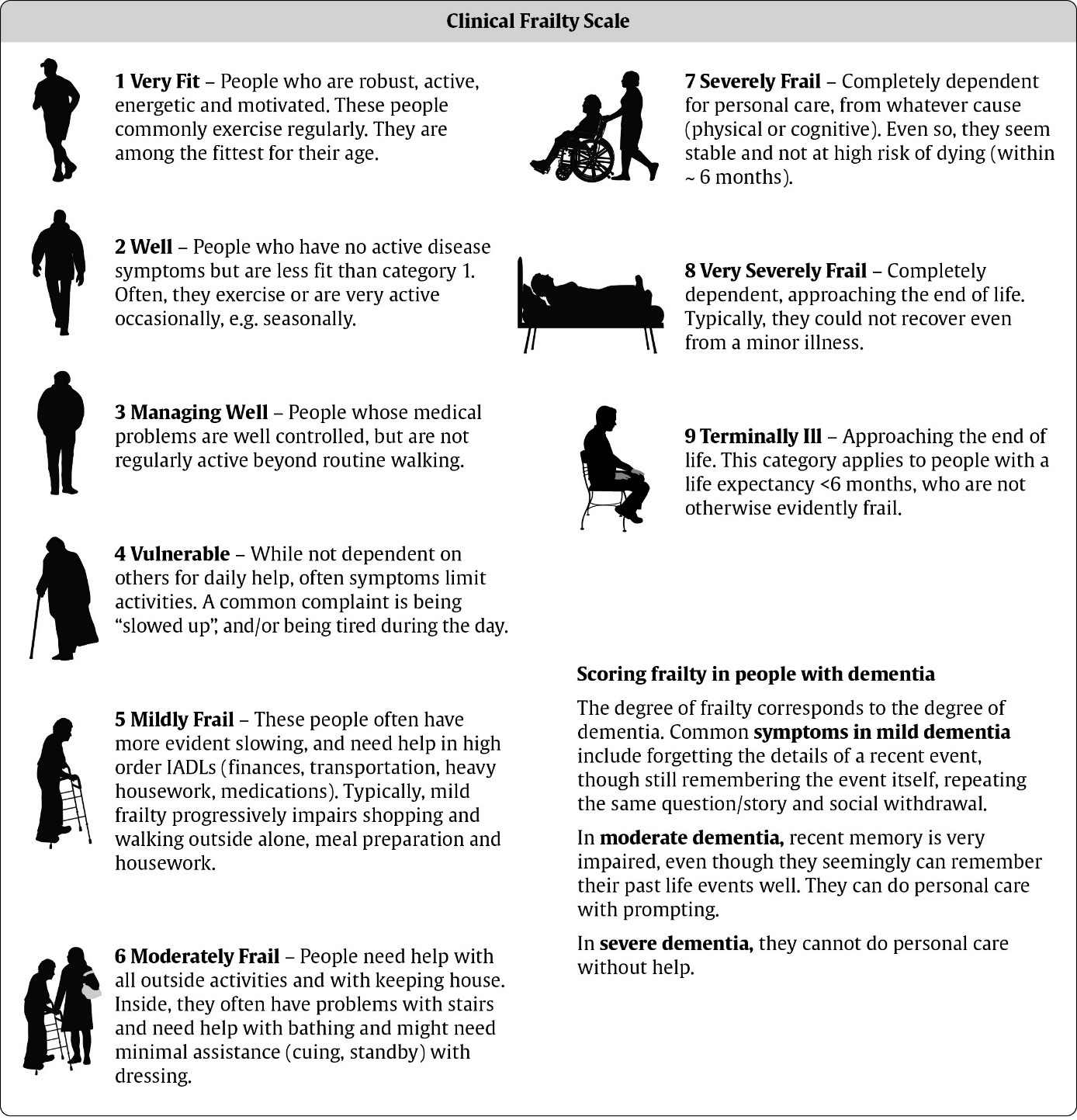


Permission to use this scale was granted from Dalhouse University, Ca, May 15 2017
