# Supplementary material for: The association of premorbid conditions with 6-month mortality in acutely admitted ICU patients over 80 years
Source: Ann Intensive Care. 2024 Mar 30;14:46. doi: 10.1186/s13613-024-01246-w (PMC10981642; doi:10.1186/s13613-024-01246-w)
Supplement: Supplementary file 5 — Additional file 5. Katz activity of daily living (ADL). [file 13613_2024_1246_MOESM5_ESM.docx]

**ESM5: Katz activity of daily living (ADL)**

| **Bathing** (Independent: 1 point, dependent: 0 point) |  |
| --- | --- |
| **Dressing** (Independent: 1 point, dependent: 0 point) |  |
| **Toileting** (Independent: 1 point, dependent: 0 point) |  |
| **Transfer** (Independent: 1 point, dependent: 0 point) |  |
| **Continence** (Independent: 1 point, dependent: 0 point) |  |
| **Feeding** (Independent: 1 point, dependent: 0 point) |  |
| *Scale from 0 (totally dependent) to 6 (independent)* | |
