# Supplementary material for: The association of premorbid conditions with 6-month mortality in acutely admitted ICU patients over 80 years
Source: Ann Intensive Care. 2024 Mar 30;14:46. doi: 10.1186/s13613-024-01246-w (PMC10981642; doi:10.1186/s13613-024-01246-w)
Supplement: Supplementary file 6 — Additional file 6. Cognitive decline questionnaire (IQCODE). [file 13613_2024_1246_MOESM6_ESM.docx]

**ESM6 : Cognitive decline questionnaire (IQCODE ) (**<https://patient.info/doctor/informant-questionnaire-on-cognitive-decline-in-the-elderly-iqcode>)

###### The IQCODE assesses cognitive decline over the last 10 years. The information is collected from caregivers.

| (check answer) | Much improved | A bit improved | Not much change | A bit  worse | Much  worse |
| --- | --- | --- | --- | --- | --- |
| 1. Remembering things about family and friends - eg, occupations, birthdays, addresses? |  |  |  |  |  |
| 2. Remembering things that have happened recently? |  |  |  |  |  |
| 3. Recalling conversations a few days later? |  |  |  |  |  |
| 4. Remembering his/her address and telephone number? |  |  |  |  |  |
| 5. Remembering what day and month it is? |  |  |  |  |  |
| 6. Remembering where things are usually kept? |  |  |  |  |  |
| 7. Remembering where to find things which have been put in a different place from usual? |  |  |  |  |  |
| 8. Knowing how to work familiar machines around the house? |  |  |  |  |  |
| 9. Learning to use a new gadget or machine around the house? |  |  |  |  |  |
| 10. Learning new things in general? |  |  |  |  |  |
| 11. Following a story in a book or on TV? |  |  |  |  |  |
| 12. Making decisions on everyday matters? |  |  |  |  |  |
| 13. Handling money for shopping? |  |  |  |  |  |
| 14. Handling financial matters - eg, the pension, dealing with the bank? |  |  |  |  |  |
| 15. Handling other everyday arithmetic problems - eg, knowing how much food to buy, knowing how long between visits from family or friends? |  |  |  |  |  |
| 16. Using his/her intelligence to understand what's going on and to reason things through? |  |  |  |  |  |

Each question is assigned from 1 to 5 points. An average of 3 points/question is normal = no change from 10 years ago
